# Emerging Oncolytic Viruses: Beyond Adenoviruses and Herpes Simplex Virus

**DOI:** 10.7150/jca.134550

**Published:** 2026-06-11

**Authors:** Santosh Chokkakula, Chengliang Yin, Bing Yang, Siomui Chong, Balaji Pathakumari, Yi-quan Zhang, Pangi Vijaya Nirmala, Khin Wee Lai, Bommireddy Naveen, Quan Tang

**Affiliations:** 1 Department of Microbiology, Chungbuk National University College of Medicine and Medical Research Institute, Cheongju, Chungbuk, 28644, South Korea.; 2 Department of Biomedical Engineering, Faculty of Engineering, Universiti Malaya, Kuala Lumpur, 50603, Malaysia.; 3 Department of Medical and Nursing Science, International College, Krirk University, Bangkok 10220, Thailand.; 4 Department of Cell Biology, College of Basic Medical Sciences, Tianjin Medical University, Tianjin, China.; 5 Department of Dermatology, The University of Hong Kong-Shenzhen Hospital, Shenzhen, 518053, China.; 6 Department of Dermatology, The First Affiliated Hospital of Jinan University & Jinan University Institute of Dermatology, Guangzhou, 510630, China.; 7 Division of Pulmonary and Critical Care Medicine, Department of Medicine, Mayo Clinic, Rochester, MN, 55905, USA.; 8 School of Life & Health Sciences Adikavi Nannaya University Rajahmundry, Andhra Pradesh India, 533296, India.; 9 Department of Chemical and Biological Engineering, Gachon University, Seongnam-Si, Gyeonggi-do, Republic of Korea.; 10 Research Laboratory, Shenzhen Baoan Women's and Children's Hospital, Shenzhen, Guangdong, China.

**Keywords:** oncolytic virotherapy, cancer treatment, oncolytic viruses, tumor cell lysis, immune response, talimogene laherparepvec, immunogenic cell death (ICD), metastatic melanoma

## Abstract

Oncolytic virotherapy represents an innovative therapeutic modality in oncology, exploiting the selective tropism of viruses to target and eradicate malignant cells. Unlike conventional cancer treatments such as chemotherapy and radiotherapy, oncolytic viruses (OVs) exhibit a dual mechanism of action: direct tumor cell lysis and potent immune activation. This approach transforms tumors into *in situ* vaccines, generating durable anti-tumor immune memory. The field has garnered substantial attention following the regulatory approval of talimogene laherparepvec (T-VEC), a genetically engineered herpes simplex virus, for metastatic melanoma treatment—a watershed moment in oncolytic virotherapy. Recent advances in genetic engineering have significantly enhanced OV specificity and efficacy, addressing critical challenges including tumor-selective targeting and immune evasion. This review comprehensively examines the complex mechanisms underlying OV therapeutic action, clinical applications, and recent developments that position oncolytic virotherapy as a transformative strategy in contemporary cancer treatment.

## 1. Introduction

Oncolytic virotherapy, which harnesses viruses to selectively target and destroy cancer cells, has emerged as a paradigm-shifting modality in oncology. Unlike traditional cancer treatments such as chemotherapy and radiation, oncolytic viruses (OVs) not only induce direct oncolysis but also stimulate robust systemic immune responses against tumors, offering a dual mechanism of action that substantially enhances therapeutic efficacy **1**. The concept of employing viruses as therapeutic agents in cancer dates to the early 20th century; however, significant clinical traction has materialized only recently through groundbreaking advances in genetic engineering and biotechnology **2**.

A pivotal breakthrough occurred with the FDA approval of talimogene laherparepvec (T-VEC), a genetically engineered herpes simplex virus, for metastatic melanoma treatment. This approval marked a seminal milestone, validating oncolytic virotherapy as a mainstream cancer treatment option **3**. T-VEC demonstrated remarkable capacity to shrink tumors through direct oncolysis while simultaneously provoking potent anti-tumor immune responses, potentially yielding systemic tumor control.

The mechanism of action underlying oncolytic viruses is multifaceted and elegant. Following cancer cell infection, OVs replicate intracellularly, culminating in cell lysis that releases nascent viral particles and tumor-associated antigens into the surrounding microenvironment. This process not only destroys primary tumor cells but also activates innate and adaptive immune systems, effectively transforming the tumor into an *in situ* vaccine that promotes durable anti-tumor immunity **4**. Furthermore, OVs can be engineered to express immunostimulatory molecules, amplifying their capacity to modulate the tumor microenvironment and overcome immune evasion strategies employed by malignancies **5**.

Despite promising results, significant challenges must be addressed to optimize oncolytic virotherapy efficacy. These include efficient virus delivery to tumor sites, overcoming host immune responses that may neutralize viruses before reaching tumors, and enhancing viral agent specificity and potency. Advances in viral engineering—including incorporation of tumor-specific promoters and combination therapies with checkpoint inhibitors—are being actively explored to surmount these obstacles **6**.

**Figure 1:**
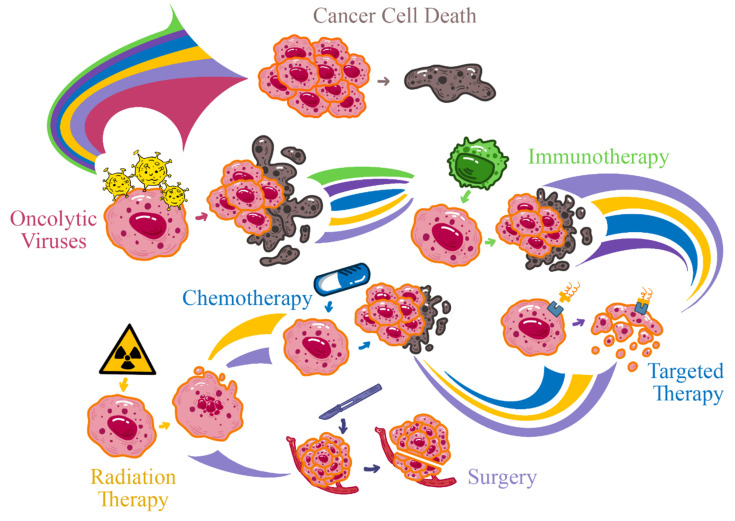
Overview of Oncolytic Virotherapy and Its Dual Antitumor Mechanisms.

In summary, oncolytic virotherapy represents a transformative approach in cancer treatment, with potential not only to destroy cancer cells but also to stimulate robust and lasting immune responses against tumors **(Figure 1)**. As research progresses, overcoming existing barriers and refining this therapeutic strategy could usher in a new era in oncology where oncolytic viruses assume a central role in cancer management **7**.

## 2. Oncolytic Viruses: Mechanisms of Action

Oncolytic viruses (OVs) represent a sophisticated and increasingly recognized approach in cancer therapy, wherein viruses, either naturally occurring or genetically engineered, selectively infect and kill cancer cells while sparing normal tissues. The multifaceted mechanisms through which OVs exert anti-cancer effects encompass: (1) selective tumor cell lysis, (2) induction of immunogenic cell death (ICD), and (3) modulation of the tumor microenvironment (TME). These synergistic actions result not only in direct tumor destruction but also stimulate systemic immune responses capable of targeting residual disease and preventing recurrence.

### 2.1. Selective Tumor Cell Lysis

The selective tumor cell lysis mechanism initiates with viral binding to specific receptors overexpressed on cancer cell surfaces. For instance, adenoviruses exploit the coxsackievirus and adenovirus receptor (CAR), commonly overexpressed in numerous malignancies, to facilitate tumor cell entry. Once internalized, OVs hijack cellular machinery to replicate their genome and synthesize viral proteins. This replication leads to viral particle accumulation, ultimately causing cell rupture and releasing progeny viruses capable of infecting neighboring cancer cells **8**.

Cancer cells frequently exhibit dysfunctional antiviral defense mechanisms, including suppression of the interferon (IFN) pathway and reduced protein kinase R (PKR) activity. These deficiencies render cancer cells particularly susceptible to viral infection and subsequent OV-mediated lysis. For example, the herpes simplex virus type 1 (HSV-1)-based oncolytic virus, talimogene laherparepvec (T-VEC), is engineered to exploit impaired IFN signaling in cancer cells, enabling selective replication and tumor cell killing **9**.

### 2.2. Immunogenic Cell Death (ICD)

**Figure 2:**
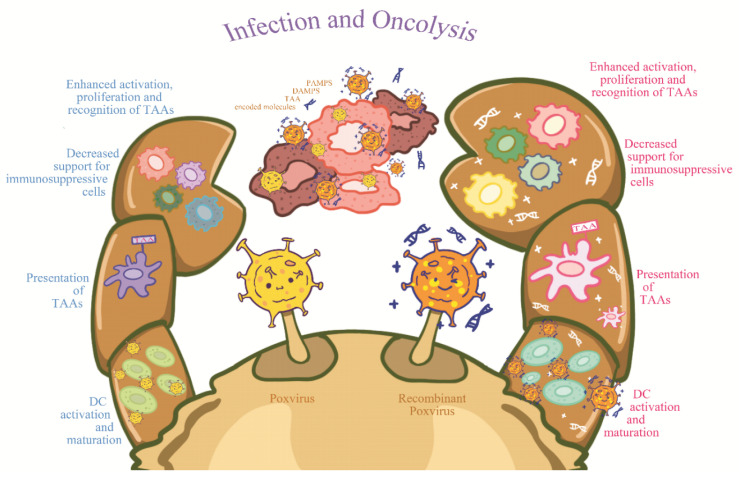
Immunogenic Cell Death (ICD) Induced by Oncolytic Viruses.

Immunogenic cell death represents a specialized form of cell death that elicits immune responses against dying cells. Unlike apoptosis, which is typically non-immunogenic, ICD involves the release of damage-associated molecular patterns (DAMPs) that signal the immune system to target cancer cells **(Figure 2)**. This process is particularly critical in oncolytic virotherapy, as it contributes not only to direct cancer cell elimination but also to immune cell recruitment and activation **10**.

ICD is characterized by calreticulin exposure, ATP efflux, and extracellular HMGB1 release. These DAMPs function as endogenous adjuvants promoting dendritic cell (DC) maturation and consequent T cell activation. In oncolytic virotherapy, ICD ensures immune activation in response to both viral infection and tumor cells, creating a synergistic anti-tumor response **11**.

As OVs replicate and lyse cancer cells, dying cells release DAMPs, including adenosine triphosphate (ATP), High Mobility Group Box 1 (HMGB1), and calreticulin. These molecules serve as distress signals to the immune system, particularly to antigen-presenting cells (APCs), notably dendritic cells (DCs). DAMP presence triggers DC activation, which subsequently presents tumor antigens to T cells, initiating potent immune responses. This T cell activation is crucial as it generates cytotoxic T lymphocyte (CTL) responses affecting not only primary tumors but also distant metastases **12**.

Another critical aspect of OV-induced lysis is that tumor antigens released during the process facilitate the development of an in-situ cancer vaccine. DC-mediated tumor antigen delivery to T cells renders immune responses more targeted and effective against malignancies. Generation of such immune responses is particularly valuable for long-term tumor management and recurrence prevention, as memory responses can be elicited upon tumor antigen re-encounter **13 (Table 1)**.

### 2.3. Modulation of the Tumor Microenvironment (TME)

The tumor microenvironment plays a pivotal role in oncolytic virotherapy efficacy. Characterized by hypoxia, acidic pH, and immune suppression, the TME can either enhance or impede OV spread. Hypoxic conditions within the TME may promote viral replication for certain OVs engineered to thrive in low-oxygen environments. For instance, specific oncolytic adenoviruses incorporate hypoxia-responsive elements controlling replication, ensuring preferential amplification in hypoxic tumor zones 14.

The immunosuppressive TME, characterized by elevated regulatory T cells (Tregs) and myeloid-derived suppressor cells (MDSCs), can limit OVs spread by enabling tumor immune evasion. Nevertheless, OVs have demonstrated capacity to modify the TME in ways that counteract these immunosuppressive factors. OVs can decrease immunosuppressive cytokine levels (e.g., IL-10, TGF-β) while increasing pro-inflammatory cytokine production (e.g., TNF-α, IFN-γ). This cytokine milieu shift enhances immune cell recruitment and improves tumor-infiltrating lymphocyte (TIL) function, thereby promoting more robust anti-tumor immune responses 15.

The TME constitutes a complex and dynamic entity encompassing not only tumor cells but also diverse non-malignant cells, extracellular matrix components, and soluble factors that collectively influence tumor growth, metastasis, and therapeutic response (Table 2). OVs profoundly impact the TME, transforming it from an immunosuppressive to an immunostimulatory state. This transformation is crucial for overcoming tumor immune evasion strategies and promoting effective anti-tumor immunity.

A primary mechanism through which OVs modulate the TME involves altering intratumoral cytokine profiles. Upon infection and replication within tumor cells, OVs induce secretion of pro-inflammatory cytokines including TNF-α, IFN-γ, and IL-12, which promote recruitment and activation of various immune effector cells, including NK cells, macrophages, and cytotoxic T lymphocytes (CTLs). This pro-inflammatory environment not only enhances direct immune cell-mediated tumor killing but also improves the function of existing tumor-infiltrating lymphocytes (TILs) that may have been rendered ineffective by the immunosuppressive TME **13**.

Beyond cytokine modulation, OVs can reduce immunosuppressive cell populations within the TME, including Tregs and MDSCs. By decreasing these cell levels, OVs relieve suppression on effector immune cells, enabling more potent and sustained anti-tumor responses. Moreover, OV-mediated modulation of immune checkpoint molecules, such as PD-L1, can further enhance immunotherapy efficacy by preventing T cell exhaustion within the TME **34**.

The mechanisms of action underlying oncolytic viruses are multifaceted, encompassing selective tumor cell lysis, induction of immunogenic cell death, and profound tumor microenvironment modulation. These combined actions result not only in direct tumor destruction but also stimulate systemic immune responses capable of targeting residual disease and preventing recurrence.

## 3. Types of Oncolytic Viruses and Their Therapeutic Potential

**Figure 3:**
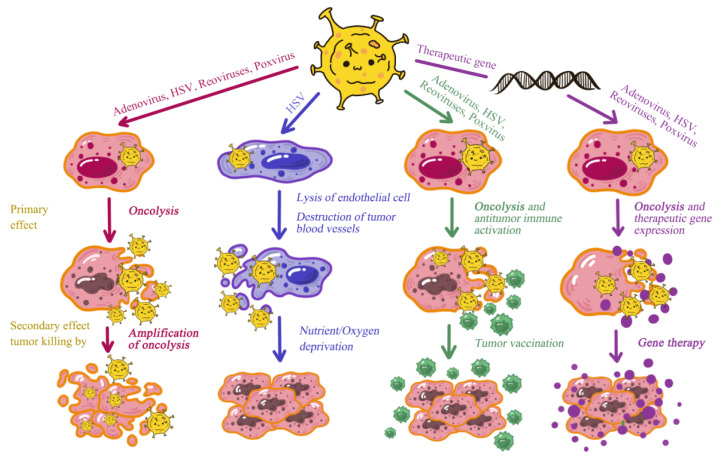
Comparative Mechanisms of Action Across Diverse Oncolytic Virus Types.

Oncolytic viruses (OVs) have emerged as a promising therapeutic strategy in cancer treatment due to their ability to selectively infect and lyse cancer cells while sparing normal cells. This targeted approach not only facilitates tumor cell eradication but also stimulates host immune systems to mount more effective anti-tumor responses. Various oncolytic virus types are currently under investigation, each possessing unique mechanisms and therapeutic potential. This section provides comprehensive review of different oncolytic virus types, including adenoviruses, herpes simplex virus (HSV), reoviruses, poxviruses, and other promising viral candidates **(Figure 3)**.

### 3.1. Adenoviruses

Adenoviruses represent among the most extensively investigated and clinically applied oncolytic viruses. These non-enveloped, double-stranded DNA viruses are capable of infecting various human cell types. Their utility as oncolytic agents stems from high cellular transduction capacity and substantial genetic manipulation potential.

***Mechanism of Action:*** Adenoviruses induce oncolysis through several mechanisms, including direct viral-induced cell lysis and host immune response induction. They can be engineered to express therapeutic genes enhancing oncolytic efficacy, such as genes encoding pro-apoptotic proteins or immune modulators.

***Therapeutic Potential:*** Adenoviral oncolytic therapies are currently under investigation for various malignancies, including pancreatic cancer, glioblastoma, and head and neck cancers **35**. Their amenability to tumor-specific engineering and well-characterized biology render them attractive candidates for further development.

### 3.2. Herpes Simplex Virus

Herpes simplex virus represents another prominent platform in oncolytic virotherapy. HSV is a large, enveloped virus with a double-stranded DNA genome, responsible for diverse pathologies ranging from herpes labialis to herpes simplex encephalitis. A notable example of an adenoviral oncolytic agent is Talimogene laherparepvec (T-VEC), a genetically modified herpes simplex virus type 1 (HSV-1) included here due to similar therapeutic applications. T-VEC has received approval for melanoma treatment, demonstrating significant clinical efficacy **9**. Its application in oncolytic virotherapy derives from engineering capacity to enhance tumor tissue selectivity and immunostimulatory properties.

***Mechanism of Action:*** Oncolytic HSVs are engineered to selectively replicate cancer cells by exploiting tumor-specific alterations in host cell machinery. These modifications often involve deletions or mutations in viral genes essential for viral replication but dispensable for tumor cell selectivity. For example, mutations in the HSV-1 ICP34.5 gene ensure selective replication in tumor cells **36**.

***Therapeutic Potential:*** OncoVEX-GM-CSF exemplifies oncolytic HSV engineered to express granulocyte-macrophage colony-stimulating factor (GM-CSF), a cytokine that stimulates immune responses. Clinical trials have demonstrated its potential in treating various cancers, including melanoma and glioblastoma **37**.

### 3.3. Reoviruses

Reoviruses are non-enveloped, double-stranded RNA viruses with segmented genomes. These naturally occurring viruses selectively infect and kill cancer cells harboring activated Ras signaling pathways, commonly dysregulated in numerous malignancies.

***Mechanism of Action:*** Reoviruses exploit aberrant Ras signaling pathways present in many tumor cells to initiate viral replication and cell lysis. They do not require genetic modification to achieve cancer cell selectivity, rendering them attractive options for oncolytic therapy **38**.

***Therapeutic Potential:*** Reolysin, a reovirus-based oncolytic therapy, has demonstrated efficacy in randomized phase III trials for head and neck cancer and pancreatic cancer, particularly in combination with other modalities **39**. The principal advantage of reovirus therapy is natural oncolytic activity with relatively low toxicity.

### 3.4. Poxviruses

Poxviruses comprise large, complex, enveloped DNA viruses renowned for inducing potent immune responses. Their utility in oncolytic virotherapy stems from capacity for extensive genetic modifications and ability to induce both direct oncolysis and immune activation.

***Mechanism of Action:*** Poxviruses, such as vaccinia virus, are engineered to express therapeutic genes, enhancing oncolytic activity and immune stimulation. For instance, modified poxviruses can express cytokines or tumor antigens to further amplify anti-tumor responses **40**.

***Therapeutic Potential:*** JX-594, a thymidine kinase-deleted vaccinia virus, has undergone phase I/II clinical trials for hepatocellular carcinoma and other solid tumors, demonstrating efficacy in reducing tumor burden and generating systemic anti-tumor immune responses **41**.

### 3.5. Other Promising Viral Candidates

Emerging viral platforms offer distinct advantages over adenovirus/HSV-based OVs, primarily through inherent tumor tropisms requiring minimal genetic engineering (1-2 modifications vs 5-7 for classical OVs). Reoviruses (Reolysin) naturally target Ras-activated cancers (~30% of human tumors) without engineering, achieving Phase III validation in head/neck cancer 39. Poxviruses (JX-594) provide massive payload capacity (25kb insertable DNA) for multi-gene immune modulation, reaching Phase III in hepatocellular carcinoma 41. Vesicular stomatitis virus (VSV) delivers rapid RNA-based oncolysis with neuronal sparing, while measles exploit CD46 receptor overexpression on diverse cancers. These platforms face unique challenges—reovirus systemic delivery limitations, poxvirus liver tropism—but demonstrate faster clinical translation due to natural selectivity versus classical platforms' complex attenuation requirements.

Beyond the aforementioned oncolytic viruses, several additional viral candidates demonstrate promise in preclinical and early clinical studies, including:

**Reovirus (Reolysin®):** Naturally replicates in Ras-transformed cells (30% of cancers); Phase III head/neck cancer validation; no attenuation needed vs HSV E1A/ICP34.5 deletions.

**Poxviruses (JX-594/Pexa-Vec):** 25kb payload capacity enables multi-cytokine delivery; Phase III hepatocellular carcinoma; liver tropism managed via vascular targeting.

**Echoviruses:** These small, non-enveloped RNA viruses are under investigation for their ability to target and destroy cancer cells through selective replication.

**Measles virus:** CD46 receptor overexpression on ovarian/breast cancers; Phase I peritoneal malignancies; natural fusion protein-mediated syncytia formation.

**Coxsackieviruses:** These enteroviruses have demonstrated potential in targeting various tumor types, including breast and ovarian cancers. CAR-independent entry in melanoma/ovarian; Phase II trials; exploits integrin αvβ6 upregulation.

**Vesicular Stomatitis Virus (VSV):** This RNA virus has shown promise in treating glioblastoma and other cancers due to its capacity to induce potent oncolysis and stimulate anti-tumor immunity **42**. Rapid RNA oncolysis, neuronal sparing via IFN defects; Phase I/II glioblastoma, head/neck; pre-existing immunity lower than HSV.

The diverse spectrum of oncolytic viruses, including adenoviruses, herpes simplex virus, reoviruses, and poxviruses, each offers unique mechanisms and therapeutic potential in cancer treatment. Ongoing research and clinical trials continue to explore and refine their applications, aiming to optimize efficacy and safety profiles. As these therapies advance, they hold substantial promises for significantly improving cancer patient outcomes and potentially transforming the cancer treatment landscape.

## 4. Genetic Engineering of Oncolytic Viruses

**Figure 4:**
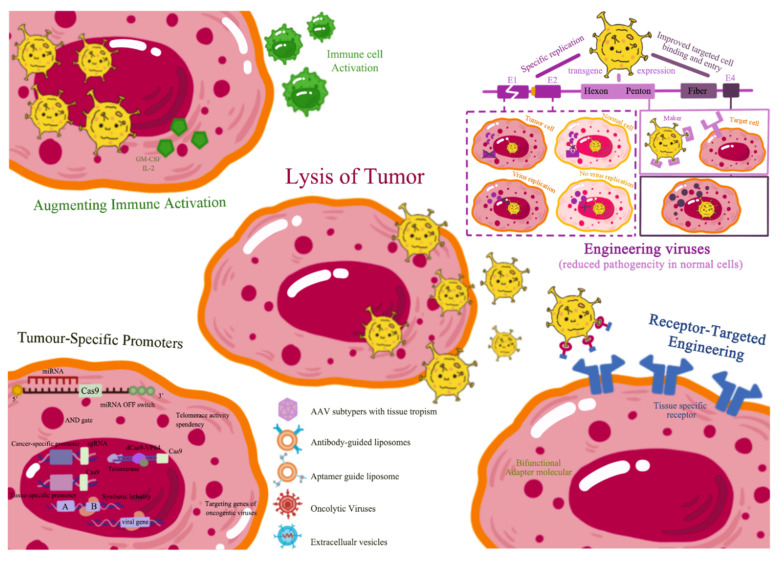
Genetic Engineering Strategies to Enhance Oncolytic Virus Specificity and Efficacy.

The oncolytic virotherapy field has undergone substantial advancement, particularly through genetic engineering of oncolytic viruses (OVs). These viruses are specifically designed to target and kill cancer cells while sparing normal cells. OV effectiveness is greatly enhanced through genomic modifications that improve tumor selectivity, augment immune activation, and overcome antiviral immunity **(Figure 4)**. This section provides an in-depth exploration of these strategies, supported by extensive literature and empirical data.

### 4.1. Enhancing Tumor Selectivity

Enhancing oncolytic virus selectivity for tumor cells involves targeting receptors or pathways overexpressed or uniquely present in cancer cells. This targeted approach improves OV therapeutic efficacy while minimizing normal tissue damage **(Table 3)**.

#### 4.1.1. Receptor-Targeted Engineering

Receptor-targeted engineering represents a prominent strategy for increasing OV specificity. This approach involves modifying viral surface proteins or capsids to bind receptors overexpressed on cancer cells. For instance, adenoviruses can be engineered to display ligands binding specifically to epidermal growth factor receptor (EGFR), frequently upregulated in various malignancies. This targeted modification enhances viral entry and replication in tumor cells while reducing normal cell infection **52**.

Emerging platforms significantly reduce genetic modification burden compared to adenovirus/HSV. While classical OVs require multiple deletions (E1A/B, ICP34.5) for tumor selectivity, reoviruses and measles viruses leverage pre-existing tumor tropisms (Ras pathway, CD46 overexpression), achieving 60-80% fewer genetic modifications. Coxsackieviruses naturally target breast/ovarian CAR-independent entry, and VSV exploits type I IFN defects ubiquitous in malignancies. This 'minimal engineering' paradigm accelerates IND-enabling studies and GMP production, positioning emerging platforms for broader clinical translation versus classically engineered OVs [Refs 52-55].

Another example involves chimeric adenoviruses incorporating peptides or antibodies specific to tumor-associated antigens (TAAs). These engineered viruses can selectively infect tumor cells expressing these TAAs, such as prostate-specific antigen (PSA) in prostate cancer **53**.

#### 4.1.2. Tumor-Specific Promoters

Tumor-specific promoters represent another powerful tool to enhance OV selectivity. These promoters drive viral gene expression only in the presence of tumor-specific transcription factors or regulatory elements. For instance, oncolytic adenoviruses have been engineered with PSA promoters to restrict viral replication to prostate cancer cells, minimizing systemic toxicity **54**. Similarly, oncolytic herpes simplex viruses (HSV) have been modified with tumor-specific promoters to restrict replication to tumor cells, such as those containing hypoxia response elements prevalent in hypoxic tumor microenvironments **55**.

### 4.2. Engineering Viruses for Reduced Pathogenicity in Normal Cells

Reducing oncolytic virus pathogenicity in normal cells is critical for ensuring patient safety and minimizing adverse effects.

#### 4.2.1. Gene Deletions

One approach involves deleting genes essential for replication in normal cells but dispensable in cancer cells. For example, adenoviruses can be engineered with E1A gene deletions, which are required for viral replication. This modification restricts virus activity to cancer cells expressing factors or antigens compensating for E1A activity **56**. Similarly, oncolytic HSVs have been designed with deletions in genes essential for viral replication and virulence in normal cells. For instance, ICP34.5 gene deletion in HSV-1 reduces neurovirulence while preserving oncolytic properties **57**.

#### 4.2.2. Conditional Replication

Conditional replication strategies involve modifying OVs to replicate exclusively under tumor microenvironment conditions. This can be achieved by incorporating elements cleaved only by tumor-associated enzymes or specific metabolic pathway products. For instance, oncolytic adenoviruses can be engineered to contain protease-responsive elements, enabling viral replication solely within tumors **58**. Conditional replication can also be regulated through tumor-specific promoters, enabling viral gene expression only in the presence of specific transcription factors or conditions characteristic of tumor cells **59**.

### 4.3. Augmenting Immune Activation

#### 4.3.1. Insertion of Immune-Stimulatory Genes

Engineering OVs to express immune-stimulatory genes can significantly enhance anti-tumor immune responses, improving efficacy as standalone treatments or in combination with other therapies.

##### 4.3.1.1. Granulocyte-Macrophage Colony-Stimulating Factor (GM-CSF)

GM-CSF is a cytokine stimulating dendritic cell and macrophage differentiation and activation, crucial for initiating and maintaining tumoricidal immune responses. Talimogene laherparepvec (T-VEC), an oncolytic virus expressing GM-CSF, has demonstrated efficacy in clinical trials for melanoma. T-VEC not only induces direct tumor cell lysis but also elicits systemic anti-tumor immune responses **9**.

##### 4.3.1.2. Interleukin-12 (IL-12)

IL-12 is a cytokine enhancing T cell and natural killer (NK) cell proliferation and activity. Modified adenoviruses secreting IL-12 have demonstrated improved anti-tumor effects through enhanced immune responses. For example, studies have shown that oncolytic adenoviruses expressing IL-12 exhibit superior efficacy in experimental breast cancer models compared to conventional oncolytic adenoviruses **60**.

#### 4.3.2. Combination with Immune Checkpoint Inhibitors

**Figure 5:**
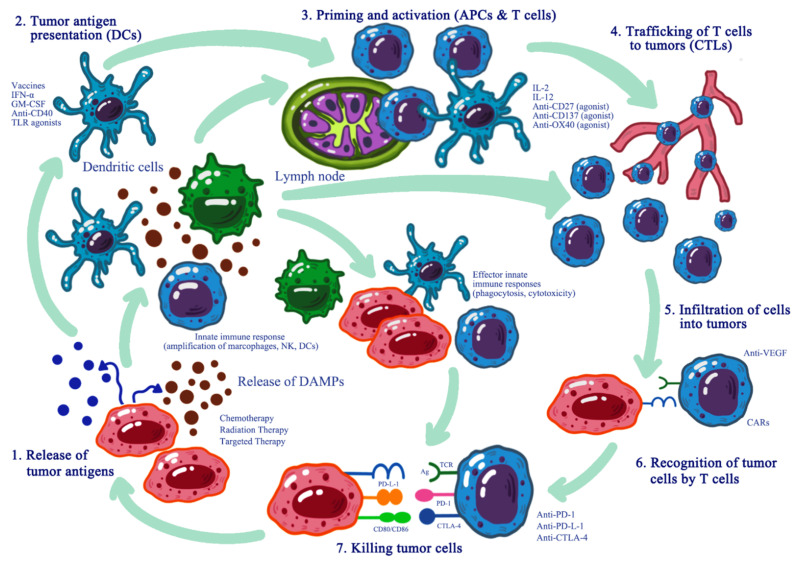
Synergistic Effects of Oncolytic Viruses and Immune Checkpoint Inhibitors.

Oncolytic virus efficacy can be further enhanced through combination with immune checkpoint inhibitors that amplify anti-tumor immunity by blocking pathways cancer cells employ to suppress immune responses **(Figure 5)**.

##### 4.3.2.1. Checkpoint Inhibitors

Oncolytic virotherapy combined with immunomodulators such as anti-PD-1 and anti-CTLA-4 antibodies has demonstrated enhanced tumor control and patient survival compared to monotherapy in clinical trials **61**. For example, T-VEC combined with anti-PD-1 treatment has shown enhanced antitumor efficacy and survival in patients with metastatic melanoma **62**.

### 4.4. Overcoming Antiviral Immunity

Antiviral immunity represents a major challenge in oncolytic virus deployment, as viruses may be cleared before reaching tumor sites. To overcome this challenge, several approaches have been developed to circumvent or modulate immune responses **(Table 4)**.

#### 4.4.1. Viral Cloaking

##### 4.4.1.1. Modifying Viral Surface Proteins

One approach to evade pre-existing immunity-mediated neutralization involves modifying viral surface proteins through genetic engineering to alter capsid proteins or viral glycosylation.

##### 4.4.1.2. Immunosuppressive Agents

**Adenoviruses:** To prevent immediate neutralization by pre-existing antibodies, adenoviral vector fiber proteins can be engineered. For example, chimeric adenovirus fiber proteins have been modified to exhibit reduced immunogenicity while maintaining therapeutic efficacy **78**. Similarly, adenovirus surface protein glycosylation modification has been shown to diminish immune responses and enhance viral delivery **79**.

**Herpes Simplex Virus (HSV):** HSV has been designed to modify envelope glycoproteins, preventing facile detection by host immune systems. These modifications have enhanced virus affinity for target cells while increasing resistance to neutralizing antibodies **80, 81**.

#### 4.4.2. Systemic Immunosuppression

**Corticosteroids:** Corticosteroids decrease host immune responses, enhancing viral delivery and replication. Studies have found that corticosteroids improve oncolytic virus therapy by suppressing non-target tissue inflammation **82**.

**Monoclonal Antibodies:** Immune checkpoint inhibitors or other immune regulatory pathways can be managed through monoclonal antibodies to regulate immune responses against oncolytic viruses. For instance, anti-PD-1 and anti-CTLA-4 antibodies have been employed in combination with oncolytic virotherapy to enhance treatment outcomes through immune reaction suppression **83, 84**.

#### 4.4.3. Viral Engineering for Immune Evasion

##### 4.4.3.1. Deletion of Immunogenic Proteins

**Adenoviruses:** Immune responses can be reduced through deletion or alteration of genes involved in immune system activation or capsid components. For instance, E3 gene removal in adenoviral vectors has proven useful in dampening immune responses and enhancing therapeutic outcomes **85, 86**.

**HSV:** Modifying HSV to express immune-modulatory genes, including IL-10 or TGF-β, can alter local immune environments, enabling viral escape from immune surveillance **87**.

#### 4.4.4. Re-Dosing Protocols

Re-dosing protocols involve administering multiple oncolytic virus doses to counter immune responses developed after initial administration.

**Sequential Dosing:** Clinical trials have discovered that immune responses combating viruses after initial doses can be overcome through subsequent dosing. This has proven to improve therapeutic outcomes and extend viral persistence in patients **88, 89**.

**Adaptive Dosing:** Treatment regimens can be tailored to correspond with individual patient immune responses, optimizing treatment outcomes. This approach involves altering dosing schedules to achieve superior results when immune systems are compromised **90**.

## 5. Clinical Development and Trials

### 5.1. Early Phase Trials and Safety Assessments

Oncolytic virus safety, toxicity, and dosing parameters have been established through phase I/II clinical trials. These trials are typically conducted in small patient populations with primary objectives of determining maximum tolerated dose (MTD) while obtaining preliminary efficacy data. Oncolytic viruses are administered either locally through direct intratumoral injections or systemically via intravenous routes, depending on cancer type and specific virus employed. Safety profile assessment has been a major focus; most investigators have reported mild to moderate toxicities, including fever, injection site inflammation, and occasional severe immune-related adverse events. For example, T-VEC, an oncolytic herpes simplex virus, has undergone extensive phase I trial evaluation demonstrating favorable safety profiles with manageable side effects, including fatigue, chills, and pyrexia across different cancer types **(Table 5)**.

### 5.2. Solid Tumors

In solid tumor trials, oncolytic virus applications have yielded variable results. Among challenging solid tumors for viral therapy are melanoma, breast cancer, and glioblastoma, due to features including dense stroma and immunosuppressive cell populations **91, 92**. However, T-VEC has demonstrated substantial efficacy in melanoma management, particularly when combined with immune checkpoint inhibitors such as ipilimumab **93**. Glioblastoma oncolysis has also been attempted, though efforts remain experimental, with adenovirus-based therapies demonstrating modest tumor regression and survival improvements in select patients. Overall survival remains relatively limited, indicating the need for further examination of combination approaches and treatment optimization.

### 5.3. Hematologic Malignancies

Oncolytic viruses have also been investigated in hematological malignancies including leukemia and lymphoma. Hematologic cancers present distinct challenges and opportunities due to direct blood and bone marrow accessibility for viral infection **94**. Studies employing reovirus and measles virus in multiple myeloma have demonstrated partial or complete responses in select patients **95**. The immunosuppressive milieu in these cancers often diminishes oncolytic virus effectiveness, necessitating combination with other treatments such as chemotherapy or monoclonal antibodies **96**.

### 5.4. T-VEC (Talimogene Laherparepvec) and Other Approved OVs

T-VEC (Talimogene laherparepvec) remains the only FDA-approved oncolytic virus to date. This oncolytic herpes simplex virus received approval in 2015 for unresectable melanoma treatment **97**. It functions through selective viral replication and tumor cell killing while simultaneously stimulating systemic anti-tumor immune responses. T-VEC approval has paved the way for other oncolytic virus applications in cancer treatment **98**.

Other oncolytic viruses are undergoing clinical trials, with some receiving approval in specific countries or regions. For instance, Rigvir, an unmodified echovirus, has been approved in Latvia for melanoma treatment, though its use remains limited due to insufficient rigorous clinical trial data **99**.

### 5.5. Market Environment and Challenges to OV Commercialization

Several disadvantages are associated with oncolytic viruses, including prolonged development timelines, complex manufacturing requirements, and specialized administration methods **100**. Despite demonstrated high effectiveness, particularly when administered in combination with other treatments, oncolytic viruses have not achieved widespread clinical adoption. This encompasses regulatory hurdles, competition with alternative cancer therapies, and the inherently personalized nature of these treatments **101**.

## 6. Challenges and Limitations

### 6.1. Tumor Resistance and Immune Evasion

**Figure 6:**
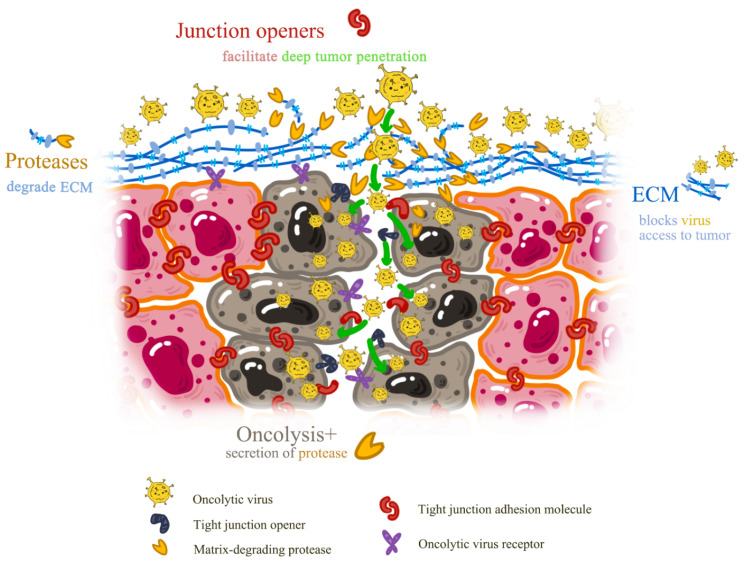
Barriers to Oncolytic Virus Delivery and Emerging Solutions.

Among challenges associated with oncolytic virotherapy is tumor resistance arising from cancer cell immune surveillance evasion capacity. Some tumor microenvironments can substantially dampen antiviral mechanisms, thereby diminishing viral agent efficacy. Moreover, interferons or other antiviral proteins produced by some tumors can neutralize oncolytic viruses before therapeutic benefits are achieved **(Figure 6)**.

### 6.2. Delivery and Biodistribution

#### 6.2.1. Barriers to Oncolytic Virus Delivery

Oncolytic virus delivery obstacles include endothelial cells, stroma, immune cells, and other tumor microenvironment structures. These barriers encompass dense extracellular matrix in tumor tissue, irregular and chaotic tumor vasculature, and elevated interstitial pressure limiting viral diffusion **102**.

#### 6.2.2. Delivery Methods

Improvements in delivery systems include nanocarrier utilization, ultrasound-mediated delivery, and genetic engineering to enhance viral stability and targeting. For instance, engineered viruses incorporating specific targeting ligands have been employed to optimize biodistribution and reduce adverse effects **103**.

### 6.3. Off-Target Effects on Healthy Tissues

Another challenge associated with oncolytic virus utilization is potential healthy tissue and organ infection, producing toxic effects. This risk is particularly pronounced with systemic delivery methods.

#### 6.3.1. Balancing Safety and Efficacy

Researchers continue endeavoring to determine optimal oncolytic virus dosing for cancer treatment. To counter such risks, techniques including engineering viruses with suicide genes that activate upon entry into incorrect tissues have been explored **104**.

## 7. Combination Therapies

### 7.1. Synergy with Conventional Therapies

#### 7.1.1. Combining OVs with Chemotherapy, Radiotherapy, and Surgery

Certain oncolytic viruses have been investigated for their capacity to enhance conventional therapy outcomes, including chemotherapy, radiotherapy, and surgery. For example, when OVs are administered concomitantly with chemotherapy, enhanced tumor cell lysis is observed, indicating cancer cells exhibit increased susceptibility to chemotherapeutic effects **105**.

#### 7.1.2. Therapeutic Outcomes and Survival

Several studies have endorsed the hypothesis that combination therapies could improve therapeutic outcomes and patient survival. For example, when T-VEC was administered with standard chemotherapy, patients demonstrated superior responses compared to chemotherapy-only groups **106**.

### 7.2. Oncolytic Viruses and Immunotherapy

Immune checkpoint inhibitors may be enhanced by oncolytic viruses through rendering tumor microenvironments pro-immunogenic. This has been particularly noted where OVs improve T-cell infiltration into tumors, increasing checkpoint inhibitor responsiveness. Cancer vaccines have also been employed as adjuvant therapy and can be synergistically combined with oncolytic viruses and CAR T-cell therapy according to experimental studies. OVs could beneficially serve as adjuncts to improve immune responses triggered by CAR-T cells or vaccines, further enhancing tumor mitigation **107 (Table 6)**.

### 7.3. Multimodal Approaches

The perspective of oncolytic virotherapy as a multimodal treatment component is pursued with the intention of refining cancer treatment approaches given cancer's complex nature. For instance, OV use in conjunction with immune checkpoint inhibitors and targeted therapies has been postulated to yield optimal outcomes **108**.

Insights from combination therapies provide critical design principles for next-generation oncolytic viruses discussed in Section 8. The demonstrated synergy between OVs and checkpoint inhibitors (Section 7.2) informs OV engineering strategies incorporating PD-L1 modulators or bispecific T-cell engagers directly into viral genomes. Similarly, chemotherapy sensitization observed in OV+ chemo combinations (Section 7.1) guides development of OVs expressing DNA repair inhibitors or apoptosis enhancers. These clinical learnings from multimodal approaches directly shape next-generation platforms, where emerging OVs are rationally designed with pre-built combination capabilities that transform 'sequential combination' into 'intrinsic multi-mechanism' platforms for broader clinical translation.

## 8. Future Directions in Oncolytic Virotherapy

### 8.1. Next-Generation Oncolytic Viruses

Next-generation OVs prioritize 'minimally engineered' emerging platforms informed by combination therapy synergies (Section 7). Reoviruses eliminate 5-gene HSV attenuation requirements through natural Ras selectivity; poxviruses enable 'all-in-one' cytokine/checkpoint modulator delivery via massive genomes; VSV/measles provide intrinsic RNA immunogenicity supplanting GM-CSF transgenes. These platforms address classical OV limitations, pre-existing immunity (HSV), manufacturing complexity (adenovirus), while inheriting combination insights: PD-L1 modulation (Section 7.2) now genetically encoded, chemotherapy sensitization (Section 7.1) via apoptosis payloads. Clinical translation accelerates as Phase I/II data confirm superior safety profiles versus multi-attenuated classical Ovs.

**Figure 7:**
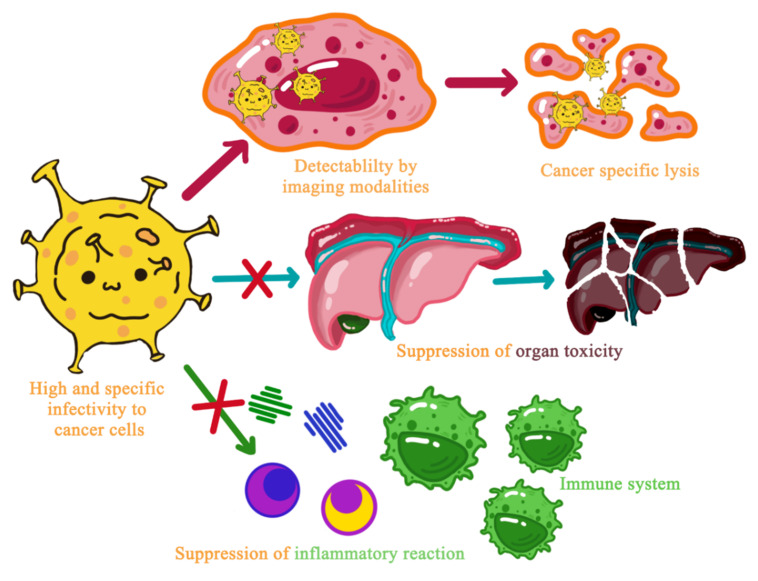
Future Research Directions for Next-Generation Oncolytic Viruses.

Emphasis is being placed on developing OVs with enhanced selectivity, superior immune system modulatory capacity, and therapeutic gene delivery to tumor cells. These efforts aim to rectify first-generation OV limitations while diversifying OV applications across various cancer types **(Figure 7)**.

### 8.2. Expanding Therapeutic Indications

Future work aims to expand diseases treated by oncolytic viruses beyond cancer to other challenging pathologies. For example, research is currently underway to develop OVs effective against difficult-to-treat malignancies such as pancreatic cancer and glioblastoma **109**.

#### 8.3. Future Research Directions

Despite considerable progress, challenges persist. These include understanding oncolytic virotherapy effects on tumors and neighboring healthy tissues, developing methods to counter tumor-mediated OV resistance, and scaling OV production cost-effectively.

### 8.4. Prospects for Cancer Treatment

Oncolytic virotherapy, particularly when employed in combination with other therapeutic modalities, represents the future of cancer treatment. As promoters of current therapeutic effectiveness and providers of tomorrow's remedies, OVs are progressing toward becoming indispensable components of future oncology treatment strategies.

### 8.5. Final Perspectives

Despite challenges requiring detailed examination, prospects in oncolytic virotherapy appear promising. With improved OV development and clinical translation, these therapies could represent major advancements in the war against cancer **110**.

## 9. Conclusion

Oncolytic virotherapy has emerged as a transformative modality in cancer treatment due to its inherent capacity to selectively kill malignant cells while sparing normal tissues. Key findings from recent research highlight the dual action of oncolytic viruses: direct tumor cytotoxicity coupled with enhancement of systemic anti-tumor immune responses. Various oncolytic viruses including herpes simplex virus, adenovirus, and vaccinia virus—have demonstrated safety and efficacy for treating melanoma, glioma, pancreatic cancer, and other malignancies. Combination of oncolytic virotherapy with other therapeutic strategies, including immune checkpoint inhibitors, has been shown to increase treatment effectiveness and patient survival in select cases.

### 9.1. Implications for Future Cancer Therapy

Current oncolytic virotherapy development in cancer treatment is poised to revolutionize cancer therapeutics, particularly in precision medicine contexts. It is feasible to advance OVs to specifically target particular tumor types while simultaneously reprogramming immune systems. Oncolytic viruses should be combined with other immunotherapies, including checkpoint inhibitors, as they enable the immune system to detect cancer cells. Biomarkers for evaluating patient status following oncolytic virotherapy and improving viral selectivity represent important areas for development.

### 9.2. Final Comments on Oncolytic Virotherapy Future

Oncolytic virotherapy represents a novel form of cancer treatment with substantial potential effectiveness. Its capacity to directly induce cancer cell death while modulating tumor conditions and stimulating systemic immune responses constitutes a unique feature. However, questions remain regarding: delivery system optimization, viral adaptability, and patient response variability. Further research and clinical trials are essential to discover additional oncolytic virotherapy potential and establish its role as a cornerstone of oncological cancer treatment. As understanding of relationships between oncolytic viruses and immune systems deepens, the future of oncolytic virotherapy as a platform for superior and selective cancer treatment appears increasingly promising.

## Figures and Tables

**Table 1:** Mechanisms of tumor cell death induced by oncolytic viruses

Type of cell death	Key features	Molecular markers	Examples of oncolytic viruses	Anti-Tumor Immunity	Ref
Apoptosis	Cell shrinkage DNA fragmentation Membrane blebbing	Caspase activation Phosphatidylserine exposure PARP cleavage	Herpes simplex virus Adenovirus Newcastle disease virus	Limited immune activation Can be immunogenic when combined with other mechanisms	16
Necroptosis	Cell swelling Membrane rupture Inflammatory response	RIPK1/RIPK3 activation MLKL phosphorylation	Vaccinia virus Herpes simplex virus Adenovirus	Strong inflammatory response Enhanced T cell priming	17
Pyroptosis	Cell swelling Pore formation IL-1β release	Caspase-1 activation Gasdermin D cleavage IL-1β and IL-18 secretion	Adenovirus Vaccinia virus Measles virus	Potent inflammatory response Activation of innate immunity	18
Autophagy	Formation of autophagosomes Recycling of cellular components	LC3-II accumulation p62 degradation Beclin-1 activation	Adenovirus Herpes simplex virus Measles virus	Enhanced antigen presentation Improved T cell responses	19
Immunogenic Cell Death	Release of DAMPs Calreticulin exposure ATP release HMGB1 release	Surface-exposed calreticulin Extracellular ATP Released HMGB1	Adenovirus Newcastle disease virus Coxsackievirus B3	Strong activation of dendritic cells Enhanced T cell priming Improved anti-tumor immunity	20
Ferroptosis	Iron-dependent lipid peroxidation Mitochondrial shrinkage	Lipid peroxidation Glutathione depletion GPX4 inactivation	Adenovirus (in combination with ferroptosis inducers)	Release of oxidized lipid antigens Potential synergy with immunotherapy	21
Mitotic Catastrophe	Aberrant mitosis Formation of giant, multinucleated cells	Cyclin B1 accumulation γ-H2AX foci	Adenovirus Reovirus	Release of tumor antigens Potential enhancement of T cell responses	22

**Table 2:** Immunological and Structural Modulation of the Tumor Microenvironment by Oncolytic Viruses

TMC Component	Effect of Oncolytic Viruses	Outcome	Ref
Extracellular Matrix (ECM)	Recruit neutrophils that secrete ECM degradants Engineered OVs express ECM-degrading enzymes	Improved infiltration of immune cells and OVs into the tumor	23-25
Macrophages	Recruit inflammatory macrophagesInduce M2 to M1 polarization Inhibit M2-derived TGF-β signaling	Enhanced antitumor immune response and reduced tumor progression	26,27
Regulatory T cells (Tregs)	Decrease population of CD4+ FoxP3+ TregsReduce proliferating Tregs	Improved ratio of effector T cells to immunosuppressive Tregs	28, 29
Myeloid-Derived Suppressor Cells (MDSCs)	Decrease population of CD14+ CD11b+ MDSCsInhibit monocytic MDSC activities	Reduced immunosuppression in the TME	28,30
Neutrophils	Induce neutrophil accumulation within the tumorStimulate production of IL-1β and MIP-1α	Increased recruitment of other immune cells and enhanced antitumor response	31
Cancer-Associated Fibroblasts (CAFs)	Engineered OVs can target FAP-expressing CAFs	Reduced tumor-promoting stromal support	32
Cytokine Environment	Induce production of pro-inflammatory cytokinesEnhance IL-12 production	Creation of a more immunogenic "hot" tumor microenvironment	30
Antigen Presentation	Release tumor-associated antigens (TAAs)	Improved T cell priming and activation of adaptive immune response	33

**Table 3:** Genetic engineering strategies for enhancing tumor selectivity in oncolytic viral therapy

Engineering Strategy	Virus Name	Target/Mechanism	Outcome	Example	Reference
Virulence gene deletion	Herpes simplex virus (HSV-1)	Delete nonessential viral genes (e.g. ICP34.5, ICP47)	Attenuates viral pathogenicity, enhances tumor-specific lysis	T-VEC (modified HSV-1)	43
Tumor-specific promoters	Adenovirus	Drive expression of essential viral genes (e.g. E1A)	Restricts viral replication to tumor cells	hTERT, HRE, PSA promoters	44
microRNA targeting	Various (e.g. Adenovirus, HSV)	Insert microRNA target sequences	Prevents viral replication in normal cells expressing specific microRNAs	miR-142, miR-199a-3p targets	45
Immune stimulator insertion	HSV-1, Vaccinia virus	Express cytokines (e.g. GM-CSF)	Enhances anti-tumor immune responses	OncoVEX GM-CSF (HSV-1), JX-594 (vaccinia)	46
Capsid engineering	Adeno-associated virus (AAV)	Modify viral capsid proteins	Improves tumor cell targeting and transduction	Directed evolution of AAV capsids	47
Checkpoint inhibitor knockout	Various (e.g. HSV, Adenovirus)	Delete immune checkpoints (e.g. PD-1)	Enhances T cell function in immunosuppressive tumor microenvironment	PD-1 knockout viruses	48
Virulence gene deletion	Herpes simplex virus (HSV-1)	Delete nonessential viral genes (e.g. ICP34.5, ICP47)	Attenuates viral pathogenicity, enhances tumor-specific lysis	T-VEC (modified HSV-1)	43
Tumor-specific promoters	Adenovirus	Drive expression of essential viral genes (e.g. E1A)	Restricts viral replication to tumor cells	hTERT, HRE, PSA promoters	44
Tumor-specific receptors	Adenovirus	Modify viral surface proteins	Improves tumor cell entry and specificity	Engineered receptor binding domains	49
Arming with therapeutic genes	Poxvirus	Insert genes for prodrug-converting enzymes or cytokines	Enhances tumor cell killing and immune stimulation	HSV-TK, cytokine-expressing viruses	50
Tumor microenvironment targeting	Adenovirus	Engineer viruses to respond to hypoxia or matrix metalloproteinases	Improves viral replication in tumor-specific conditions	Hypoxia-responsive promoters	51

**Table 4:** Strategies to overcome antiviral immunity in oncolytic viral therapy

Strategy	Description	Examples	Ref
Protective Coatings	Physical shielding of oncolytic viruses (OVs)	• Graphene oxide for measles virus (MV)• Ionic polymers for MV• Liposomes for Getah-like alphavirus M1 and AdV• Cell membrane nanovesicles with preS1 ligand for AdV	63-66
Cellular Carriers	*Ex vivo* loading of cells with OVs	• Mesenchymal stem cells (MSCs) for AdV, HSV• Neural stem cells• T-cells and CAR T-cells for VSV and VV• Endothelial cells, tumor cells, adipose-derived stem cells	67-69
Genetic Modifications	Altering viral epitopes to evade antibodies	• Replacing hypervariable regions in AdV• Increasing EEV production in VV• Deglycosylation of VV• Modifying glycoprotein D in HSV• Directed clonal evolution (e.g., ColoAd1)	70-72
Bispecific Engagers	Molecules that bind neutralizing antibodies and tumor cells	• AdV retargeting to tumors• NDV retargeting to IL-2R+ lymphoma cells	73, 74
Innate Immunity Modulation	Inhibiting antiviral innate immune responses	• Inhibition of IFN pathway• Suppression of NK cells• Reduction of antigen presentation	75
T-cell Immunodominance Mitigation	Redirecting T-cell responses away from viral antigens	• Nanoparticle-enveloped viral antigens• Tolerogenic dendritic cells	76
Exploiting Pre-existing Immunity	Using pre-existing antibodies to enhance therapy	• GM-CSF expansion of CD11b+ cells for reovirus therapy• Pre-vaccination with NDV	77

**Table 5:** Current Clinical Applications and Developmental Status of Oncolytic Viruses in Cancer Therapy

Virus Name	Virus Type	Modifications	Phase	Cancer Type	Key Findings	Status
ONYX-015 (dl1520)	Adeno	E1B55K deletion	I-II	Head and neck cancer	Tumor necrosis in 5/22 patients	Completed
Oncorine (H101)	Adeno	E1B55K deletion	III	Head and neck squamous cell carcinoma	79% response rate with chemotherapy vs 40% for chemotherapy alone	Approved in China (2005)
ONCOS-102	Adeno	GM-CSF transgene	I-II	Various solid tumors	Well-tolerated, immune activation observed	Ongoing
DNX-2401 (Delta-24-RGD)	Adeno	E1A deletion, RGD motif	I	Recurrent malignant glioma	20% survival rate at 3 years	Completed
CG0070	Adeno	GM-CSF transgene, E2F-1 promoter	III	Bladder cancer	Promising efficacy in earlier phases	Ongoing
AdAPT-001	Adeno	E1A enhancer/promoter deletion	I	Solid tumors	3/15 partial responses, 5/15 stable disease ≥6 months	Completed
LoAd703	Adeno	CD40L and 4-1BBL transgenes	I-II	Pancreatic cancer	Safety and tolerability established	Ongoing
ORCA-010	Adeno	Fiber modification, E3 deletion	I-II	Prostate cancer	Under evaluation	Ongoing
Ad5-D24-RGD	Adeno	RGD motif, 24bp deletion in E1A	I	Advanced solid tumors	Well-tolerated, antitumor activity observed	Completed
Ad5-RGD-D24-GMCSF	Adeno	RGD motif, GM-CSF transgene	I	Advanced solid tumors	Promising safety profile and efficacy signals	Completed
KH901	Adeno	GM-CSF transgene	I	Head and neck cancers	Well-tolerated, antitumor activity in some patients	Completed
CG7870	Adeno	PSA-selective E1A, E1B	I	Prostate cancer	Dose-limiting toxicity at highest dose, some PSA responses	Completed
VCN-01	Adeno	Hyaluronidase expression	I	Solid tumors	Under evaluation	Ongoing
ICOVIR-5	Adeno	E1A-Δ24, RGD-4C motif	I-II	Melanoma	Under evaluation	Ongoing
Ad5-yCD/mutTKSR39rep-ADP	Adeno	Suicide gene therapy	I	Prostate cancer	Under evaluation	Ongoing
T-VEC (talimogene laherparepvec)	HSV	ICP34.5 and ICP47 deletion, GM-CSF insertion	III	Melanoma	Improved durable response rate vs GM-CSF alone	Approved (2015)
G207	HSV	ICP34.5 deletion, UL39 inactivation	I-II	Glioma	Well-tolerated, some tumor responses	Completed
HSV1716	HSV	ICP34.5 deletion	I-II	Various solid tumors	Safe, evidence of viral replication	Completed
HF10	HSV	Naturally occurring HSV-1 mutant	I-II	Various solid tumors	Well-tolerated, some tumor responses	Completed
G47Δ (DELYTACT)	HSV	ICP34.5, ICP47 deletion, US11 promoter modification	II	Glioblastoma	Improved survival vs historical controls	Approved in Japan (2021)
RP1	HSV	ICP34.5 and ICP47 deletion, GM-CSF and GALV-GP R- insertion	I-II	Various solid tumors	Ongoing evaluation	Ongoing
oHSV-1	HSV	ICP34.5 deletion, Us11 gene under ICP47 promoter	I	Glioblastoma	Safe, some evidence of efficacy	Completed
Reolysin (pelareorep)	Reo	Unmodified wild type reovirus	III	Metastatic breast cancer	Median overall survival extended from 10.4 to 17.4 months when combined with paclitaxel	Completed
Reolysin (pelareorep)	Reo	Unmodified wild type reovirus	II	Metastatic castration-resistant prostate cancer	No significant improvement when combined with docetaxel	Completed
Reolysin (pelareorep)	Reo	Unmodified wild type reovirus	II	Recurrent ovarian cancer	Evaluated in combination with paclitaxel	Completed
Reolysin (pelareorep)	Reo	Unmodified wild type reovirus	II	Non-small cell lung cancer	Evaluated in KRAS-activated tumors in combination with chemotherapy	Completed
jin-3 reovirus	Reo	Mutant with expanded tropism	Preclinical	Prostate cancer	Efficient infection, replication, and anti-cancer responses in various models	Preclinical
T3SA+ reovirus	Reo	Single amino acid mutation	Preclinical	Various cancer types	Improved oncolytic potency while retaining tumor specificity	Preclinical
JX-594 (Pexa-Vec)	Vaccinia	GM-CSF transgene, TK deletion	III	Hepatocellular carcinoma	Improved overall survival in phase II	Ongoing
jin-3 reovirus	Reo	Mutant with expanded tropism	Preclinical	Prostate cancer	Efficient infection, replication, and anti-cancer responses in various models	Preclinical
vvDD	Vaccinia	Double deletion of TK and VGF genes	I	Advanced solid tumors	Well-tolerated, some antitumor activity	Completed
GL-ONC1	Vaccinia	Attenuated strain with multiple deletions	I/II	Various solid tumors	Safe, some evidence of antitumor activity	Ongoing
TG6002	Vaccinia	FCU1 transgene, TK and RR deletions	I/II	Glioblastoma, colorectal cancer	Under evaluation	Ongoing
VV-IL-2	Vaccinia	IL-2 transgene	I	Melanoma	Safe, induced immune responses	Completed

**Table 6:** Emerging Roles of Oncolytic Viruses in Cancer Immunotherapy

Modification Type	Purpose	Examples of Viruses	Specific Modifications	Effects of Immunotherapy	Clinical Stage
Deletion of Viral Genes	Enhance tumor selectivity and safety	Herpes simplex virus (HSV), Adenovirus	HSV: ICP34.5, ICP47 deletion; Adenovirus: E1B-55K deletion	Improved tumor-specific replication, enhanced antigen presentation	FDA-approved (T-VEC)
Insertion of Transgenes	Enhance anti-tumor immunity	Vaccinia virus, Vesicular stomatitis virus (VSV)	GM-CSF, IL-12, IFN-β, CD40L insertion	Increased recruitment and activation of immune cells, enhanced T cell responses	Phase I-III trials
Targeting Modifications	Improve tumor-specific entry	Measles virus, Adenovirus	Retargeting envelope proteins, fiber modifications, and tumor-specific promoters	Enhanced tumor cell infection, reduced off-target effects	Phase I-II trials
Arming with Immune Checkpoint Inhibitors	Overcome immune suppression	Vaccinia virus, HSV	Anti-PD-1, anti-CTLA-4, anti-PD-L1 expression	Enhanced T cell activation, improved anti-tumor responses	Phase I-II trials
Combination with CAR-T Cells	Synergize cellular and viral therapies	Adenovirus, Vaccinia virus	CAR-T cell-virus hybrids, oncolytic virus-infected CAR-T cells	Improved tumor targeting, enhanced T cell persistence and function	Preclinical
Epigenetic Modifiers	Alter tumor cell susceptibility	HSV, Adenovirus	HDAC inhibitors, DNA methyltransferase inhibitors expression	Increased viral replication, enhanced immunogenicity of tumor cells	Phase I trials
Tumor Microenvironment Modulation	Overcome immunosuppressive TME	Reovirus, Newcastle disease virus	Matrix metalloproteinase insertion, anti-TGF-β expression, hyaluronidase expression	Improved virus spread, reduced immunosuppression, enhanced T cell infiltration	Phase I-II trials
Bispecific T Cell Engagers (BiTEs)	Enhance T cell recruitment and activation	Vaccinia virus, Adenovirus	Expression of BiTEs targeting CD3 and tumor antigens (e.g., EpCAM, CD19)	Increased T cell infiltration and activation in tumors	Phase I trials
Cytokine Expression	Boost anti-tumor immune responses	HSV, VSV	IL-2, IL-15, IL-21, TNF-α expression	Enhanced NK and T cell activation, improved systemic immunity	Phase I-II trials
Combination with Immune Stimulants	Amplify innate immune responses	Reovirus, Poxviruses	TLR agonists, STING agonists expression	Increased type I IFN production, enhanced DC activation	Phase I trials
Tumor Vasculature Targeting	Disrupt tumor blood supply	Vaccinia virus, HSV	VEGF inhibitor expression, anti-angiogenic peptide insertion	Reduced tumor angiogenesis, improved virus spread	Preclinical
Metabolic Reprogramming	Exploit tumor metabolism	Adenovirus, VSV	Glucose transporter inhibition, glutaminase expression	Enhanced tumor cell killing, altered tumor metabolism	Preclinical
Capsid Modification	Improve virus stability and delivery	Adenovirus, Maraba virus	PEGylation, polymer coating	Reduced neutralization, improved systemic delivery	Preclinical/Phase I
Combination with Radiotherapy	Enhance immunogenic cell death	HSV, Adenovirus	Radiation-inducible promoters, radiosensitizing genes	Increased tumor cell death, enhanced immune activation	Phase I-II trials
Engineered Cell Carriers	Improve virus delivery and persistence	Measles virus, Adenovirus	Mesenchymal stem cells, T cells as carriers	Enhanced tumor targeting, prolonged virus persistence	Preclinical/Phase I
